# Binding interaction of a ring-hydroxylating dioxygenase with fluoranthene in *Pseudomonas aeruginosa* DN1

**DOI:** 10.1038/s41598-021-00783-9

**Published:** 2021-10-29

**Authors:** Shu-Wen Xue, Yue-Xin Tian, Jin-Cheng Pan, Ya-Ni Liu, Yan-Ling Ma

**Affiliations:** 1 Shaanxi Provincial Key Laboratory of Biotechnology, Key Laboratory of Resources Biology and Biotechnology in Western China, Ministry of Education, College of Life Science, Northwest University, Xi’an, 710069 Shaanxi China; 2 College of Life Science, Northwest University, 229 Taibai North Rd, Xi’an, 710069 Shaanxi China

**Keywords:** Microbiology, Structural biology, Environmental sciences

## Abstract

*Pseudomonas aeruginosa* DN1 can efficiently utilize fluoranthene as its sole carbon source, and the initial reaction in the biodegradation process is catalyzed by a ring-hydroxylating dioxygenase (RHD). To clarify the binding interaction of RHD with fluoranthene in the strain DN1, the genes encoding alpha subunit (RS30940) and beta subunit (RS05115) of RHD were functionally characterized through multi-technique combination such as gene knockout and homology modeling as well as molecular docking analysis. The results showed that the mutants lacking the characteristic alpha subunit and/or beta subunit failed to degrade fluoranthene effectively. Based on the translated protein sequence and Ramachandran plot, 96.5% of the primary amino-acid sequences of the alpha subunit in the modeled structure of the RHD were in the permitted region, 2.3% in the allowed region, but 1.2% in the disallowed area. The catalytic mechanism mediated by key residues was proposed by the simulations of molecular docking, wherein the active site of alpha subunit constituted a triangle structure of the mononuclear iron atom and the two oxygen atoms coupled with the predicted catalytic ternary of His_217_-His_222_-Asp_372_ for the dihydroxylation reaction with fluoranthene. Those amino acid residues adjacent to fluoranthene were nonpolar groups, and the C_7_-C_8_ positions on the fluoranthene ring were estimated to be the best oxidation sites. The distance of C_7_-O and C_8_-O was 3.77 Å and 3.04 Å respectively, and both of them were parallel. The results of synchronous fluorescence and site-directed mutagenesis confirmed the roles of the predicted residues during catalysis. This binding interaction could enhance our understanding of the catalytic mechanism of RHDs and provide a solid foundation for further enzymatic modification.

## Introduction

Polycyclic aromatic hydrocarbons (PAHs) have troubled various ecosystems because of their prolonged persistence, potential mutagenic and carcinogenic properties^1,2^. High molecular weight PAHs containing four or more fused aromatic nucleus have attracted widespread attention since they are recalcitrant in the environment^3,4^. Among those, fluoranthene with four fused aromatic nucleus was commonly acted as a model compound of HMW PAHs for biodegradation researches in view of its structural similarity with other carcinogenic HMW PAHs^5–7^. Compared with physicochemical treatment techniques, bioremediation is considered to be an efficient and promising strategy for PAHs removal^8–10^. In the degradation of PAHs by aerobic bacteria, dioxygenases that catalyze the introduction of two atoms of molecular oxygen into an organic substrate play a central role. Generally, dioxygenases have been divided into distinct groups, ring-hydroxylating dioxygenases (RHDs) that are involved in the oxidation of aromatic compounds to produce dihydroxy non-aromatic intermediates, and ring-cleaving dioxygenases that catalyze the cleavage of the aromatic nucleus^5,11,12^. The degradation mechanisms of fluoranthene were demonstrated in different bacterial strains, but are mainly initiated by the dihydroxylation of the aromatic ring^13,14^. In most previous studies, the fission of fluoranthene ring was first acted at C_1_–C_2_, C_2_–C_3_, C_7_–C_8_ and C_9_–C_10_ positions by dioxygenase^5–7,15^.

For potential applications in environmental protection, it is important to understand the interaction mechanism between RHD and substrate, since the structural features of substrates may affect substrate preferences and the region-specificity^16–18^. RHDs from PAH-degrading bacteria reported are compared in accordance with their substrate specificity, genetic organization, and sequence relatedness^19–21^. So far, more than 1300 RHDs have been deposited in the NCBI database and identified on account of sequence similarity, including naphthalene dioxygenases from *Pseudomonas* sp. NCIB9816-4 and *Rhodococcus*sp. NCIMB 12038, cumene dioxygenase from *Pseudomonas fluorescens* IP01, nitrobenzene dioxygenase from *Comamonas* sp. JS765, biphenyl dioxygenase from *Rhodococcus* sp. RHA1, and carbazole-1,9 α-dioxygenase from *Pseudomonas resinovorans* CA10^14,22–26^. RHDs are multicomponent enzymes comprising of an oxygenase associated with the specific electron carriers, with the alpha subunit containing a Rieske-type [2Fe-2S] cluster and a mononuclear iron center at the active center. The alpha subunit of the terminal oxygenase contains the catalytic site of the dioxygenation reaction^13,27,28^. In most cases, RHDs share common structural properties, including quaternary structure and conserved residues in the Rieske and catalytic domains of the alpha subunit that are involved in binding of the metal centers^14,28^. However, a large space in the active site may support the existence of diversiform substrate binding mode. Most of all, their manipulation, to effectually increase substrate specificity and enhance specific activity, is dependent on an understanding of substrate binding-site. Nevertheless, the structural determinants responsible for substrate recognition remain unclear, which greatly hinders the utilization of enzyme.

In previous study, we reported that *Pseudomonas aeruginosa* DN1 harbored more than 100 candidate genes involved in PAHs degradation (such as *rhd*), and the fluoranthene degradation was initiated by dioxygenation at the C_1_–C_2_, C_2_–C_3_ and C_7_–C_8_ positions respectively^6^. But the binding interaction between RHD and fluoranthene is still unknown, thus, the present study was to elucidate the potential interaction mode between fluoranthene and the active sites of RHD using gene knockout approach combined with homology modeling and molecular docking, with an aim to uncover structural characteristics of RHD and its region-specificity towards fluoranthene for further providing enzyme modification strategies.

## Results

### Growth curves and fluoranthene degradation efficiencies of the wild-type strain DN1 and the deficient mutants

**Figure 1:**
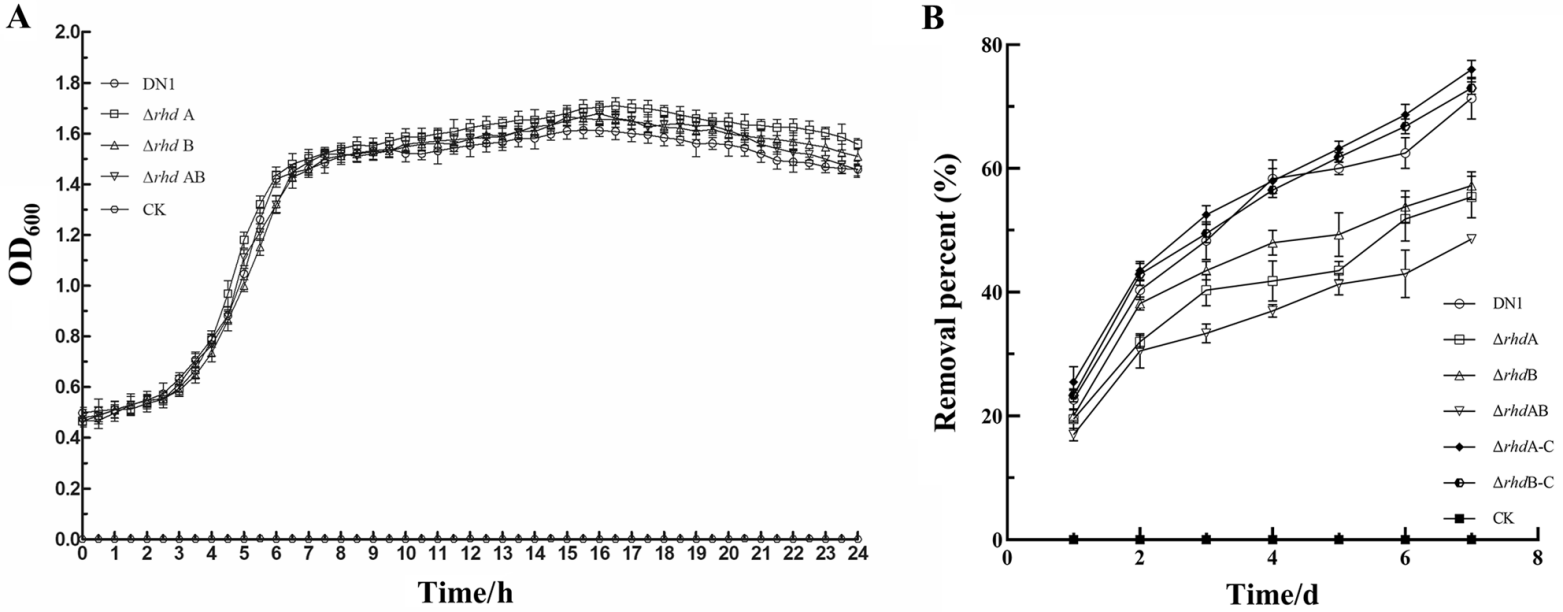
Growth curves in LB medium (**A**) and removal percent during growth period of the strain DN1, and its mutants and the complemented strain (△*rhd*A-C and △*rhd*B-C) in the presence of 50 μg/ml fluoranthene (**B**). The values presented are the average ± standard deviation of experiments run in triplicate. The open symbols stands for growth curves of those strains, and the filled symbols stands for the removal percent of fluoranthene, respectively. CK represented the cultures without cell inoculum in (**A**) and cultures containing fluoranthene without inoculum in (**B**). These samples were used as controls.

To characterize the role of alpha subunit and beta subunit of RHD during the process of fluoranthene degradation, single-knockout mutants of alpha and beta subunits and double-knockout mutants were constructed in this study (see Supplementary Fig. S1 online). The growth curves of the wild-type DN1 strain and its mutants were measured at OD_600_ at regular intervals in LB medium, and their degradation potential to fluoranthene was monitored in MSM medium containing 50 μg/mL fluoranthene with the concomitant changes of consumption for 7 days cultivation (Fig. 1). As shown in Fig. 1A, cells of the wild-type DN1 strain started multiplying after a 1 h incubation period and reached the exponential growth phase within 3 h. The mutants grew at a rate similar to that of the wild-type strain. The degradation efficiency analysis of the wild-type strain and its mutants using GC analysis revealed that the wild-type strain was the most efficient strain in removing fluoranthene, and it degraded more than 71% of 50 μg/mL fluoranthene within 7 days (Fig. 1B). The single-knockout mutants of alpha and beta subunits were inferior to the wild-type strain with a 55 ~ 57% fluoranthene-degradation efficiency, while the degradation rate of the double-knockout mutant was much lower (48%) than that of the DN1. This meant that alpha subunit (RS30940) and beta subunit (RS05115) of RHD were co-functional in the removal of fluoranthene, as the mutants showed different degradation rates. The mutant in absence of alpha subunit had a comparable lower utilization rate than did the beta subunit deletion. Together with the previous reports that alpha subunit of RHD was responsible for the initial dihydroxylation reaction of PAHs, these data suggest that alpha subunit played a major role during the process of fluoranthene degradation^14,22–26^. However, it was observed that the oxidization of aromatic substrates by RHD was not the only way to degrade fluoranthene by the strain DN1, since double-knockout mutants still retained the ability to utilize fluoranthene. This implied that a compensatory mechanism was responsible for the regulation and coordination of these multi-step catalytic reactions in the degradation of fluoranthene, which was consistent with our previous reports that plenty of genes and gene clusters in the strain DN1 could contribute to the degradation of aromatic compounds^6,29^.

### Structural exploration of active center of alpha subunit

According to previous studies that the initial step of fluoranthene utilization was performed by the alpha subunit of RHD^14,22–26^, the nucleotide sequence of the alpha subunit (RS30940) was obtained from the whole genome sequence of the wild-type DN1 strain and translated into the amino acid residues by the software DNAStar5.0, wherein the alpha subunit comprised 428 amino acids. Homology modeling was accomplished on the basis of the crystal structure of the template 4QUR, a crystal structure of stachydrine demethylase with molecular weight of approximately 49.8 kDa from *Sinorhizobium meliloti* 1021 (https://doi.org/10.2210/pdb4QUR/pdb) that displayed a high structural similarity to the alpha subunit. Then the modeled crystal structure of the alpha subunit was examined by means of Ramachandran plot to analyze the distribution rationality of the amino acid residues^30^. Meanwhile, 3VCA (http://www.rcsb.org/structure/3VCA) from *Sinorhizobium meliloti* 1021 having an identity of 94.71% with 4QUR was used to verify the validity of homology modeling of the modeled crystal structure of the alpha subunit^31^.

**Figure 2:**
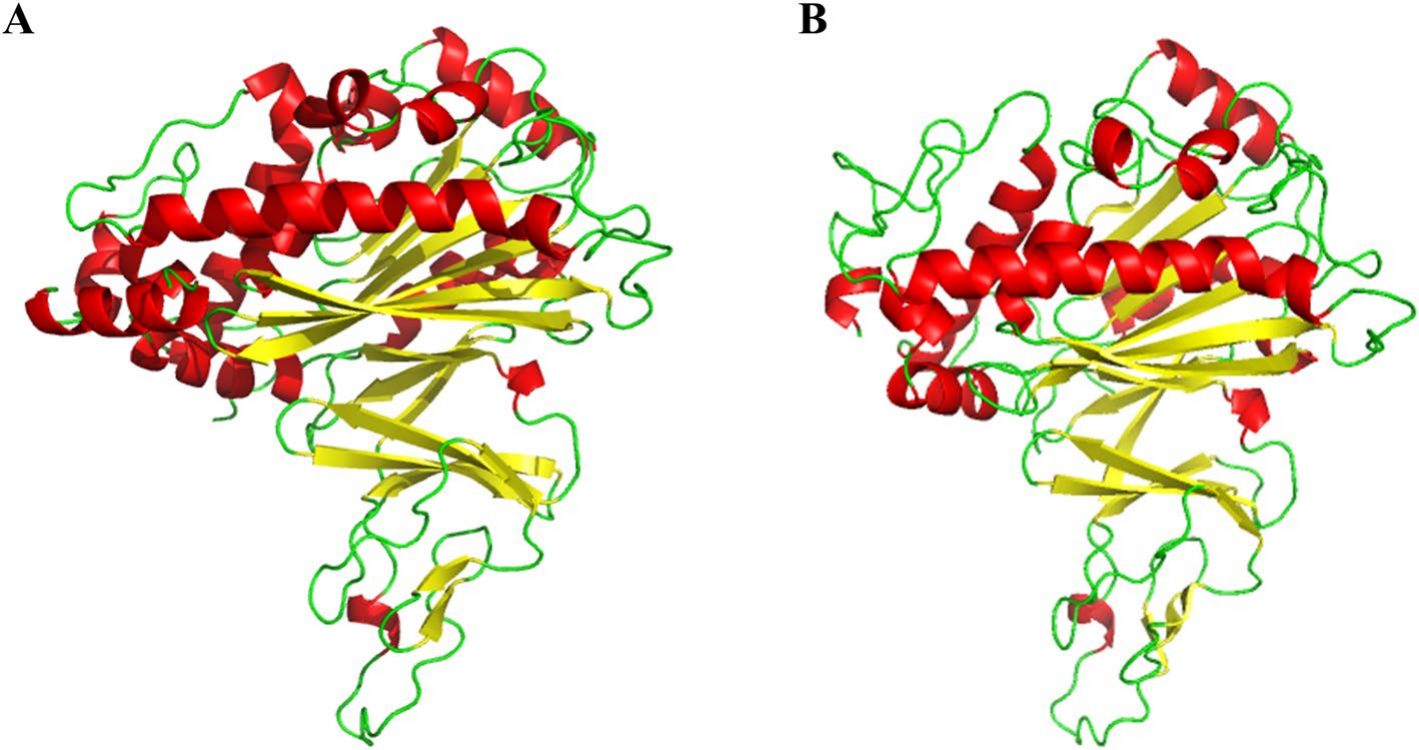
The schematic of modeled crystal structure of RHD alpha subunit using a Ramachandran plot (*Shading* with *different colors* in the diagram represent different secondary structures. *Red colors* represent α helix, *yellow colors* represent β sheet, and *green colors* represent random coil) for the homology modeling according to the crystal structure of the template 4QUR (**A**) and 3VCA (**B**).

The resulting amino acid sequence alignment verified that the primary structure sequences of the alpha subunit of the strain DN1 and template 4QUR had a similarity of 47.6% (see Supplementary Fig. S2 online), which was in accordance with studies that have confirmed that homologous proteins own an analogous backbone structure with the template when the recognition of amino acid sequences is above 30%^32,33^. The BLAST analysis certified that not only the crystal structure of the template was appropriate for the homology modeling by SWISS-MODEL, the crystal structure of 3VCA was also applicable for the homology modeling by SWISS-MODEL. Then the modeled structure of the alpha subunit was examined by Ramachandran plot, showing that 96.5% of the amino acid residues were in the permitted region, 2.3% in the allowable zone, and 1.2% in the outlier area (Fig. 2). Based on superposition and comparison analysis of the crystal structure of the template, the alpha subunit of RHD was predicted to have a catalytic domain containing mononuclear iron that had a fold predominant by an antiparallel β-pleated sheet against which helices were occupied (see Supplementary Fig. S3 and S4 online).

The difference for the major amino acid residues around the active site of the templates and the modeled enzymes might be a pivotal factor resulting in the variation of the catalytic abilities and metabolic pathways in disparate bacterial strains, and these residues were compared in this study shown in Table 1. Cys202 and Ala223 residues in the template were replaced by Ile209 and Leu230 respectively in the modeled alpha subunit. The hydrophilic residues Glu201, Asp219, and Arg200 in the template were replaced by the hydrophobic residues Val208, Leu226, and Leu207 respectively, increasing the hydrophobic property of the active site. Therefore, the active site residues of the alpha subunit of RHD in the wild-type strain DNI were significantly disparate from that of the template 4QUR. These replacements meant that a majority of the amino acid residues in the substrate binding-site were hydrophobic, thus, augment in the nonpolar cavity of the RHD alpha subunit was beneficial for binding of fluoranthene. Distinctions observed in the amino acid residues of active site among dioxygenases may briefly explain differences in substrate specificity and catalytic efficiency of the enzymes from various sources^34,35^. So far, many aromatic-hydrocarbon degraders from the genus *Pseudomonas* have been reported to have displayed diverse PAH-degradation ability, and their metabolism mechanisms have been elucidated^6,19,21,36–38^. The amino acid sequences of RHD alpha subunit from the strain DN1 showed a maximum identify of 93.92% with that from other *Pseudomonas* strains (https://blast.ncbi.nlm.nih.gov/Blast.cgi, see Supplementary Fig. S5 online), indicating that the strain DN1 was different with other strains from the genus *Pseudomonas*, while the homology between them was rather limited. This might give rise to the variety of the biological functions of this strain.

**Table 1:** Structural comparison of the major amino acid residues around the active sites of the template 4QUR and target enzyme alpha subunit.

Template enzyme	Target enzyme alpha subunit
Cys202	Ile209
Ala223	Leu230
Glu201	Val208
Asp219	Leu226
Asn198	Trp205
Arg200	Leu207
His307	Pro314
Asp360	Ala367
His204	Asn211
Trp196	Ala203
Asn208	Cys215
Leu218	Leu225
Val222	Leu229
Gly229	Thr236
Thr304	Leu311
Pro310	Trp317
His312	His319
Asp358	Trp365

### Binding interaction of fluoranthene with active site of alpha subunit

**Figure 3:**
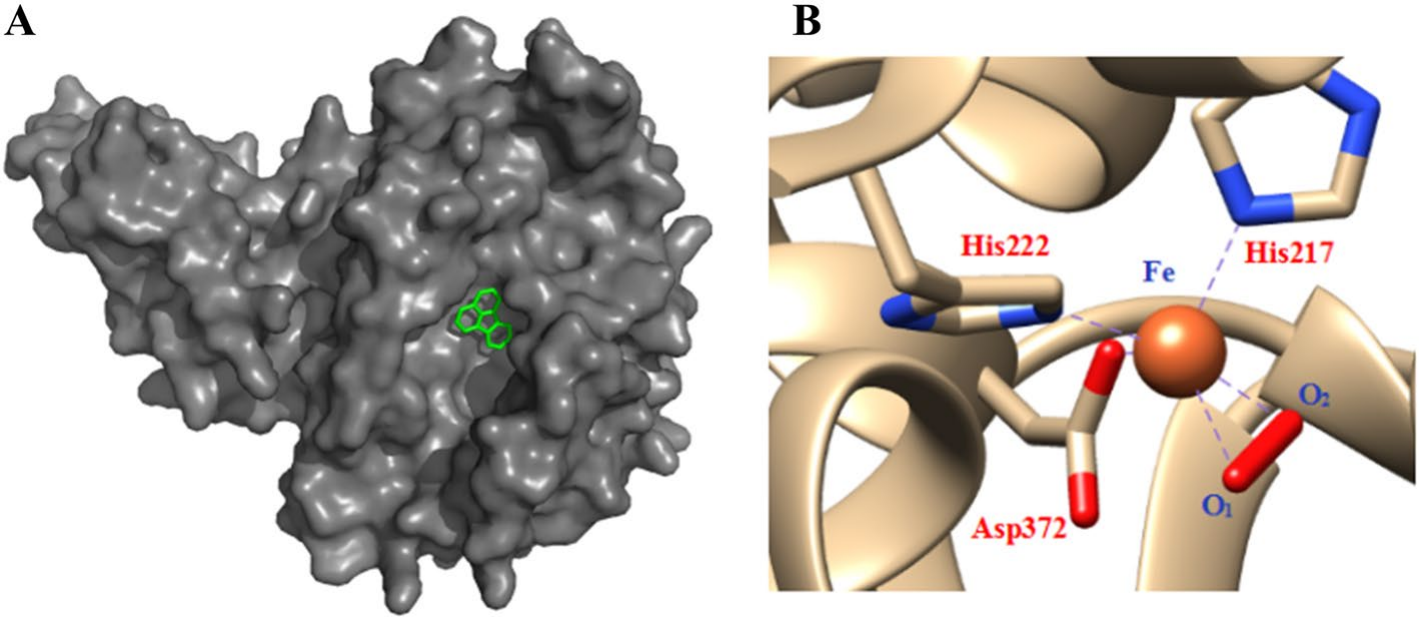
The combination state of fluoranthene in the active site of RHD (**A**: fluoranthene structure was shown in stick mode and colored *green*) and the composition of the active site in RHD alpha subunit (**B**: a triangle structure of the mononuclear iron atom and the two oxygen atoms combined with a catalytic ternary of His_217_-His_222_-Asp_372_).

The active site of the alpha subunit interacting with the substrate is indispensable for elucidating the catalytic mechanism of enzymes interaction with substrates in a high accuracy^39^. For molecular docking, fluoranthene was used as the substrate and reached into the cavity of the modeled alpha subunit of RHD after 100 docking runs based on the most frequent locus and the lowest energy, suggesting a good binding interaction between fluoranthene and RHD alpha subunit (Fig. 3A). As displayed in Fig. 3B, the two oxygen atoms and the mononuclear iron atom comprised a triangular structure, where the iron atom at the catalytic site was appropriated to accept the substrate fluoranthene, as well as coordinated with conserved residues including His_222_, His_217_, and Asp_372_ for oxygenation of substrates^40–42^. These binding forces played a key role in the oxidation of fluoranthene to give cis-dihydrodiol or cis-diol-carboxylic acid by the strain DN1 due to the direct co-ordination of O_2_ to the Fe (II) center. As known for existing dioxygenases, the transition metals, such as iron, serve as catalytic site that are regulated by a conserved 2-His-1-carboxylate triad, and the alpha subunit consists of a hydrophobic pocket with a mononuclear Fe (II) center that acts as the substrate binding-site^35,43–46^. That is, the ferredoxin-binding site is served as a restrain on the surface of the oxygenase trimer at the adjacent interface to alpha subunits. A similar signature of depression has been observed between the alpha and beta subunits of naphthalene dioxygenase, confirming that it might be the binding-site for ferredoxin in this hexameric oxygenase^30,47^.

**Figure 4:**
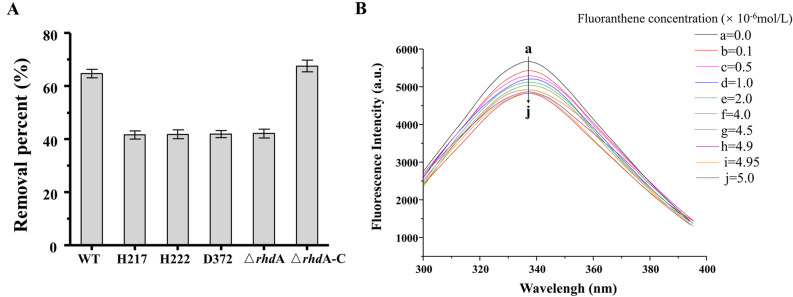
Degradation efficiency of wild-type DN1 and its mutants RHD aplha subunit His217 (H217), -His222 (H222), -Asp372 (D372) and alpha subunit (△*rhd*A), and the complemented strain (△*rhd*A-C) in the presence of 50 μg/ml fluoranthene (**A**). The values presented are the average ± standard deviation of experiments run in triplicate. Fluorescence quenching spectra of RHD alpha subunit under different fluoranthene concentrations (a–j) at 298 K (**B**).

To confirm that predicted residues by the simulation of molecular docking indeed played crucial roles during fluoranthene degradation, the amino acids of in the active site of alpha subunit gene sequence were site-directed mutated into Ala, respectively. Compared to the removal percent of the wild type DN1, a similar tendency to alpha subunit deficient strain was observed in the mutants His_217_, His_222_, and Asp_372_ (Fig. 4A). In addition, the His-tag RhdA was purified and detected through Western Blotting and SDS-PAGE (see Supplementary Fig. S6 online), and the fluorescence quenching technique was used to analyze the interactions of fluorantyhene with RHD alpha subunit. The fluorescence spectrum of purified alpha subunit showed a maximal emission peak at 337.4 nm when the excitation wavelength was 278 nm. With an addition of fluoranthene from 1.0 × 10^−7^ to 50.0 × 10^−7^ mol/L, the peak intensity decreased gradually without any obvious shift (Fig. 4B). Furthermore, the Stern–Volmer plots of the fluorescene quenching of RHD alpha subunit by fluoranthene at different temperatures with the corresponding parameters were shown in Supplementary Fig. S7 and Supplementary Table S1^48,49^. Those results indicated that the intrinsic fluorescence of RHD alpha subunit could be quenched by fluoranthene and the quenching mechanism should be static quenching, providing a direct evidence for the interaction of fluoranthene with alpha subunit. It was therefore confirmed that these residues predicted by molecular docking simulation indeed played decisive roles during catalysis.

**Figure 5:**
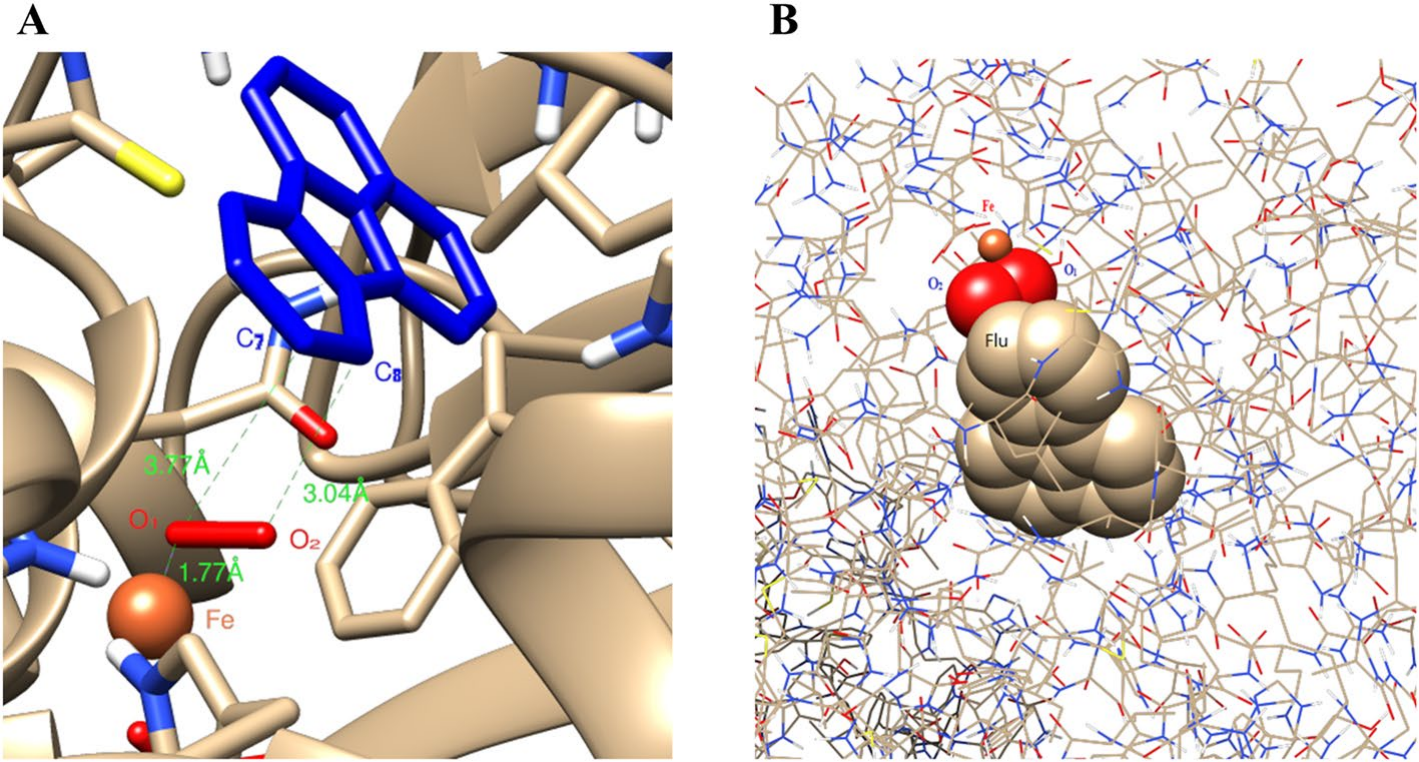
The detailed interaction between fluoranthene and the active site of alpha subunit (**A**: the fluoranthene structure was shown in stick mode and colored *blue*, and the positions of C_7_ and C_8_ atoms on the fluoranthene ring were the best oxidation sites in the active activity. **B**: the oxygen atoms, iron atom and fluoranthene were shown in ball mode).

**Figure 6:**
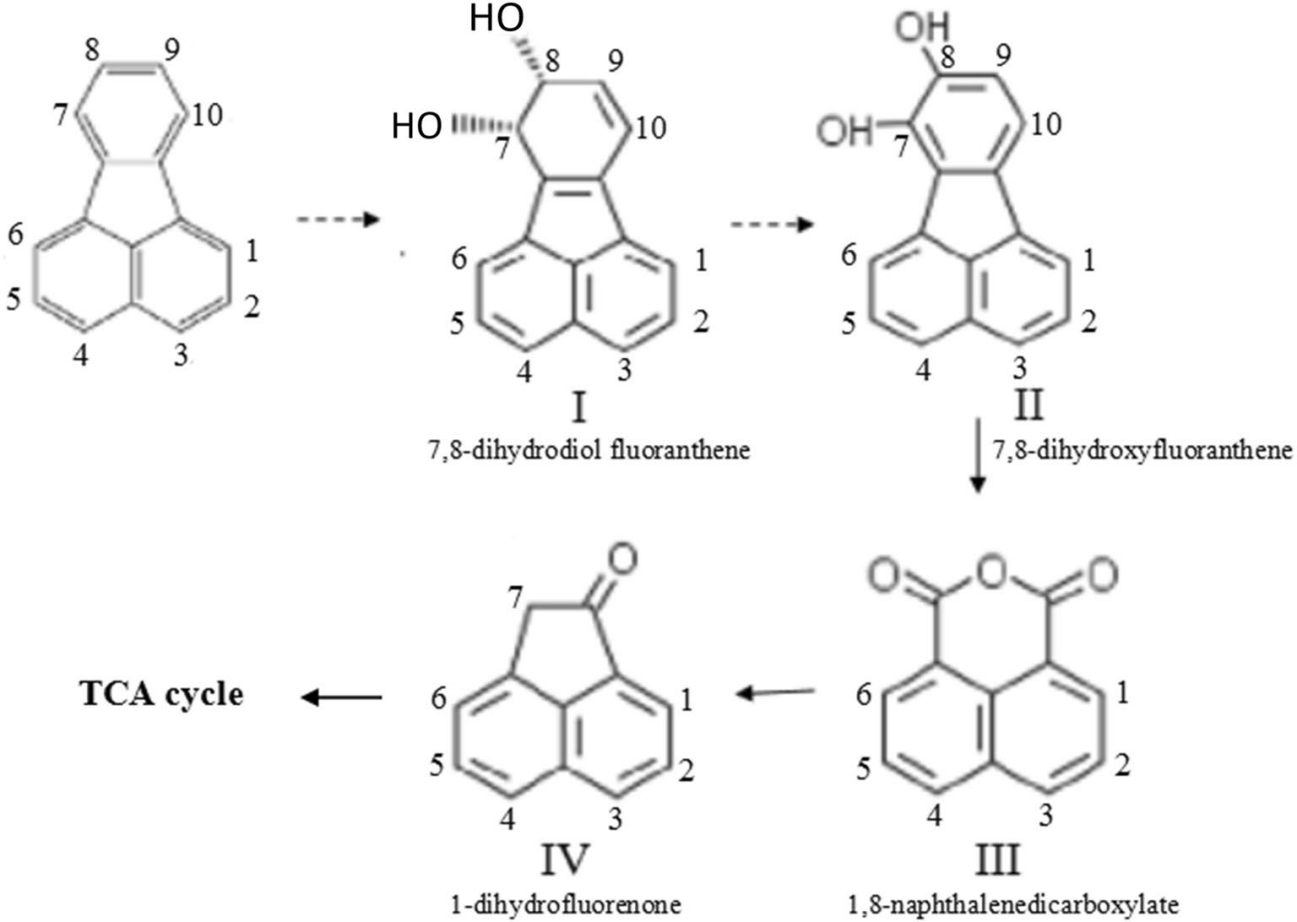
A proposed degradation pathway of fluoranthene in the strain DN1. The *dotted arrow* represents a putative degradation reaction based on RHD, and the *solid arrow* suggesting the degradation step was deduced by the analysis of the previous detected metabolites (He et al. 2018).

Meanwhile, the binding interaction of fluoranthene with the catalytic site of alpha subunit in detail was shown in Fig. 5, which displayed that the distances between C_7_-O and C_8_-O were 3.77 Å and 3.04 Å respectively, and both of them were parallel (Fig. 5A). The mode of RHD binding with fluoranthene indicated that fluoranthene fitted in the substrate-binding pocket with carbons 7 and 8 of the ring proximal to mononuclear iron might be the optimal oxidation positions in the catalytic center cavity, since they were much closer to the two oxygen atoms compared with the other carbon atoms on fluoranthene ring (Fig. 5B). The resulting model allowed to deduct that fluoranthene would fit the ligand binding pocket in RHD alpha subunit. Therefore, the initial hydroxylation of fluoranthene in the strain DN1 was concluded to occur at the C_7_-C_8_ positions and form intermediate for the next cycle of the reaction, as shown in Fig. 6. Following the binding with fluoranthene, the dioxygen molecule was activated for the formation of the binary peroxide complex that subsequently reacted with the carbon–carbon double bond of fluoranthene at C_7_–C_8_ positions to form fluoranthene *cis*-7, 8-dihydrodiolas in the formation process of the product. This best docking pose displayed the predicted orientation of substrate for the highest binding affinity, which were consistent with a previous metabolite analysis that shown that fluoranthene underwent a reaction catalyzed by an RHD at the C-7,8-position, thus, validating this theoretical study^6^.

## Discussion

RHDs are crucial for PAHs degradation by aerobic bacteria, catalyzing the initial oxidation step by introduction of atoms of O_2_ to formation of a cis-dihydrodiol and controlling the degradation rate^5,11,14^. The attack on different carbon positions indicates alternate pathways for biotransformation of PAHs and region-specificity of RHDs related to the metabolism properties of PAHs-degrading bacteria^5–7,15^. Besides, interactions of substrates with active center residues are regarded as controlling the orientation of the substrate relative to the active site and ultimately the regio- and enantio-selectivity of the catalytic reaction, which was in utmost consistent with the region-selectivity of the hydroxylation reactions^22,24^.

In general, the large size of its substrate-binding pocket and the flexibility of residues located at the entrance of dioxygenases, resulted in exceptionally broad substrate specificity and its ability oxidize large PAH molecules^16–18^. Based on the molecular docking analysis, the exact binding sites of the RHD alpha subunit in the DN1 were identified with a catalytic ternary of His_217_-His_222_-Asp_372_ in active site of RhdA, proposing that the interaction mode between the residues in the active site and the ligand can control the binding orientation of substrate^22,24,40–42^. The present structure represented a valuable frame to investigate the role of certain residues on the substrate specificity and/or catalytic activity of the enzyme through site-directed mutagenesis. The result of fluorescence quenching technique showed that the intrinsic fluorescence of RHD alpha subunit could be quenched by fluoranthene, providing a direct evidence for the interaction of RHD alpha subunit with fluoranthene^48,49^. To summarize, our experimental and molecular modeling data showed that the binding interaction of the fluoranthene ring with RHD alpha subunit in the DN1 through inserting an oxygen atom into the methyl C-H bond, which was reported minimally about region-specificity of RHDs related to the metabolism properties of *Pseudomonas aeruginosa*, to the best of our knowledge.

Taken together, these findings indicated that the large size and the particular topology of the substrate-binding pocket of the alpha subunit were the most crucial element to satisfy for accommodation of fluoranthene, which provided an approving explanation for the possible binding behavior between substrate and the active center of RHD^22,34,35^. To better understand the underlying steric constraints of the whole RHD, we need to resort to a comprehensive approach in further research, such as a X-RAY study on the crystal structure of RHD, which would systematically characterize the large size and particular topology of its catalytic pocket and provided the basis for the study of its reaction mechanism.

## Conclusions

In this study, alpha subunit of RHD of *P. aeruginosa* DN1 was acquired to play a pivotal role in the catabolism of fluoranthene using gene knockout approach. In addition, a visual interaction of fluoranthene with the active center of the alpha subunit was obtained and analyzed by means of homology modeling and molecular docking, which displayed that C_7_-C_8_ positions were the most suitable positions for fluoranthene dihydroxylation. The results of synchronous fluorescence and site-directed mutagenesis revealed obvious changes in the microenvironment for the initial hydroxylation of fluoranthene, which provides a better understanding of the underlying molecular mechanisms of fluoranthene degradation by RHD in the strain DN1.

## Materials and methods

### Bacterial strain and media

*P. aeruginosa* DN1 (CP017099) used herein has been previously reported to display strong ability to utilize fluoranthene as its sole carbon source^6,29^.

Luria–Bertani and mineral salt medium (MSM, pH 7.2) were used for the growth of the bacterium. MSM included the following ingredients per liter: 1.0 g (NH_4_)_2_SO_4_, 5.0 g K_2_HPO_4_, 3.0 g KH_2_PO_4_, 0.5 g NaCl, 0.5 g MgSO_4_·7H_2_O, 0.01 g NaNO_3_, and 0.005 g FeSO_4_·7H_2_O with a pH of 7.2. To examine the utilization of fluoranthene by the strain DN1 and its mutants, fluoranthene was first dissolved in ethyl alcohol to form a 10 mg/mL solution, and the solution was then added to the MSM medium to gain given concentrations^50^. We confirmed that ethyl alcohol was neither toxicant to the DN1, nor was it degraded by the strain. Fluoranthene (99%) was purchased from Aladdin Industrial Inc. (Shanghai, China).

### Deletion and complementation of rhdA

Coding sequence of alpha subunit (RS30940) and beta subunit (RS05115) of RHD was obtained from the whole genome sequence of the strain DN1. To determine the function of these subunits, single- and double-knockout mutants of the two genes were constructed using a *Sac*B-based strategy^51^. According to our previous studies^6,50^, to construct △*rhd*A, the null mutant of alpha subunit, polymerase chain reactions (PCR) were performed to amplify sequences upstream and downstream of the targeted deletion gene. The upstream fragment was amplified using the primer pair, pEX-*rhd*A-up-S and pEX-*rhd*A-up-A, and the downstream fragment was amplified with the primer pair, pEX-*rhd*A-down-S andpEX-*rhd*A-down-A (Table 2). The PCR products were digested by endonuclease (TaKaRa, Japan), cloned into the *Bam*HI/*Hind*III digested gene replacement vector pEX18Ap, and the recombinant plasmid pEX18Ap-*rhd*A was obtained. The plasmids were then electroporated into the strain DN1 with selection for carbenicillin resistance. Colonies with both carbenicillin resistance and loss of sucrose (10%) susceptibility were selected on Luria Bertani agar plates containing 300 μg/mL of carbenicillin and 10% sucrose, which typically indicate a double-crossover event and gene replacement occurrence. The mutated strain △*rhd*A was further confirmed by PCR. Gene disruption of the beta subunit and both the subunits together was achieved by a similar strategy. For complementation, the *rhd*A fragment was amplified from chromosomal DNA of DN1 with the primers *rhd*A-F and *rhd*A-R (Table 2). The obtained DNA fragment was ligated into a Shuttle expression vector (pAK1900), and the recombinant vector was transformed into mutant strain Δ*rhd*A, resulting in complemented strains Δ*rhd*A -C.

**Table 2:** Primers used by this research.

Primers	Sequences(5´ to 3´)	Application
*rhd*A-up-S	CTCAAGCTTCCTTTCCTGCCGTTCCTG	Constructing *rhd*A mutant
*rhd*A-up-A	AGCTCTAGACAACTCCGACGCCTACCAG	
*rhd*A-down-S	GCATCTAGACAGGCGCTGGTCGCAGTA	
*rhd*A-down-A	TATGGATCCTCGCTACCTGGCGCTGAC	
*rhd*A- Sense	TCCCACCTTAGTCCTTTTGCC	
*rhd*A-Antisense	CATTTACCTGCCAGCGACAT	
*rhd*B-up-S	TACGGTACCTGGAGCATGTCGGTAAGGC	Constructing *rhd*B mutant
*rhd*B-up-A	GCCTCTAGAGTAACTTGCGTCGTCGGTCT	
*rhd*B-down-S	TCGTCTAGAGAGCCGTCTTCAACCATCGC	
*rhd*B-down-A	TACAAGCTTCATGCTCATCCGGTCCTTGC	
*rhd*B- Sense	CGCCACCAACTATGTGCTACG	
*rhd*B- Antisense	GCCGATGAACGAGATCAACCC	
Pr-*rhdA-S*	GGGgatatgATGGACGTCACTTCCACCCTGAGTC	Expression of RhdA protein
Pr-*rhdA-A*	CCGgaattcGCTGCCGGCCACCTTGCG	
*rhdA*-F	ATGtctagaGATGATCTCTCCGCGCGCACG	Site-directed mutagenesis
*rhdA*-R	TACaagcttTTAGCTGCCGGCCACCTTGCG	
*rhd*A -His217-F	CGCGAGTGCTACGCCTGCAACGGCTCC	
*rhd*A -His217-R	GGAGCCGTTGCAGGCGTAGCACTCGCG	
*rhd*A -His222-F	ACGGCTCCGCCCCGGAACTG	
*rhd*A -His222-R	CAGTTCCGGGGCGGAGCCGT	
*rhd*A -Asp372-F	ACCAGGCTCGTCGGCTGGC	
*rhd*A -Asp372-R	GCCAGCCGACGAGCCTGGT	

The mutation site of *rhd* A is underlined and the mutation of nucleotide bases, all of which are translated to alanine residues.

### Test of the DN1 and its mutants in MSM supplemented with fluoranthene

The wild-type DN1 strain and the mutant strains △*rhd*A, △*rhd*B, and △*rhd*AB were grown in LB medium and inoculated in MSM supplemented with 50 μg/mL fluoranthene as the sole carbon source at 30 °C and 200 rpm for 7 days, respectively. The initial OD_600_ was 0.15, and flasks without cell inoculum were used as blanks to assess the abiotic loss. OD_600_ values in LB medium were taken every interval time to plot resulting growth curves. The fluoranthene degradation efficiency of the wild-type DN1 strain and its mutants were determined from the loss of substrate from each liquid culture. Cultures without fluoranthene and those containing fluoranthene without inoculum were used as controls. All the cultures were taken out at different time points to estimate cell concentration and fluoranthene residue using gas chromatography (GC)^52^. All measurements were repeated thrice, and the results shown were the average values of three replicates, along with the standard errors.

### Interaction analysis of fluoranthene and active center of alpha subunit

To analyze the interaction between fluoranthene and the alpha subunit of RHD, the nucleotide sequence of alpha subunit was translated to amino acid sequence by the software DNAStar5.0^30^. The translated protein sequence was used to obtain protein modelling of alpha subunit of RHD by homology modeling. A published crystal structure of 4QUR (http://www.rcsb.org/structure/4QUR) was selected as the template for homology modeling by the automatic search function in SWISS-MODEL, and the modeled structure was examined by Psi/Phi Ramachandran plot^53–57^. To elucidate the binding mechanism and binding site between fluoranthene and the alpha subunit of RHD, molecular docking was done using the software AutoDock^58^.

### Site-directed mutagenesis

Based on the molecular docking analysis, the predicted key residues in the *rhd*A gene sequence were further confirmed by site-directed mutated into Ala, respectively^59^. The site-directed mutagenesis of *rhd*A was achieved through overlap PCR with the primers (Table 2). Briefly, primer *rhd*A-F and primer His217-R and primer *rhd*A-R and primer His217-F were used to amplify the *rhd*A gene by PCR with cycling conditions as following: 98 °C for 10 s, 64 °C for 15 s, 72 °C for 30 s or 1 min (30 cycles), respectively. The cleaned PCR products were then added into a PCR mixture as the templates and run for 10 cycles without primers (98 °C for 10 s, 62 °C for 15 s, 72 °C for 1 min). After that, the primer His217-F and primer His217-R were added to repeat this process for 30 times under the same condition and then further elongated at 72 °C for another 5 min. The final fragments were sequenced after cloning into a Shuttle expression vector (pAK1900), and the recombinant vector was transformed into strain △*rhd*A, giving the distinctive mutant strain. The mutation of His222 and Asp372 to Ala was achieved by a similar strategy.

### Preparation and purification of recombinant RhdA protein

To obtain the recombinant RhdA protein, the genome of the DN1 was used as template to amplify *rhd*A fragment with primer pair Pr-*rhd*A-S and Pr-rhdA-A (Table 2). The PCR products were digested by endonuclease (TaKaRa, Japan) at 37 °C for 5 h, and then cloned into the *Nde*I/*Eco*RI restriction endonuclease digested pET-28a and subsequently transformed into *E. coli* BL21 Star (DE3). The recombinant was screened on LB-Kan agar plate and further confirmed by DNA sequencing. Strain E. coli BL21 (pET28a-*rhd*A) cultured overnight was then inoculated into 1 L LB-Kan medium with 220 rpm shaking at 37 °C until OD600 reached 0.6 ~ 0.8. Then, IPTG was added to the culture for another 4 h shaking at 37 °C. Cells were harvested by centrifugation (8000 g for 10 min at 4 °C), and washed twice with water. The pellets were then resuspended in 20 mL buffer A (10 mM Tris, 500 mM NaCl, 20 mM imidazole, pH 8.0) and broken via ultrasonic cell crushing apparatus. After binding with 5 mL pre-equilibrated Ni SepharoseTM excel (GE Healthcare) at 4 °C for 6 ~ 8 h, the buffer A was used to remove miscellaneous protein and buffer B (10 mM Tris, 500 mM NaCl, 500 mM imidazole, pH 8.0) was used to obtain higher purity protein. The eluted protein was dialyzed (20 mM Tris, 200 mM NaCl) and then detected by SDS-PAGE.

### Fluorescence measurement

The fluorescence quenching technique was used to analyze the interactions of fluoranthene with RHD alpha subunit since it contained 10 residues of tryptophan in the primary structure, of which Trp_317_ and Trp_365_ was present around the active site^60^. Experimental systems were prepared as follows: 2.5 mL of phosphate buffer (pH 7.5) containing 5.0 × 10^−6^ mol/L RHD alpha subunit and different concentrations of fluoranthene solutions (0–50.0 × 10^−7^ mol/L) were successively added in a series of 15 mL colorimetric tubes. The prepared sample solutions were mixed thoroughly and equilibrated before fluorescence measurement. The fluorescence spectra of the samples were measured using an F-4500 spectrofluorometer (Hitachi, Japan) with 1.0 cm quartz cell. The fluorescence emission spectra of samples were recorded in the wavelength range of 265 − 500 nm at 298, 304, and 310 K, respectively. The spectra of fluoranthene solutions without enzyme additions were measured as a blank to eliminate background signal. Synchronous fluorescence spectra of RHD alpha subunit in the absence and presence of fluoranthene were measured at 298 K by setting the excitation and emission wavelength interval at 15 and 60 nm, respectively. The spectra were recorded in the wavelength range of 200 − 400 nm.

## Supplementary Information

Supplementary Information.
