# Supplementary material for: Binding interaction of a ring-hydroxylating dioxygenase with fluoranthene in Pseudomonas aeruginosa DN1
Source: Sci Rep. 2021 Oct 29;11:21317. doi: 10.1038/s41598-021-00783-9 (PMC8556375; doi:10.1038/s41598-021-00783-9)
Supplement: Supplementary file 1 — Supplementary Information. [file 41598_2021_783_MOESM1_ESM.pdf]

Supplementary Table S1 Stern-Volmer quenching constants of RHD alpha subunit fluorescence system at different temperatures (pH = 7.5, λex = 278 nm)

Temperature (K)	K_{SV} (L/mol)	Kq (L/mol/s)	R^2
298	6.45×10^4	6.45×10^{12}	0.9976
304	5.80×10^4	5.80×10^{12}	0.997
310	5.51×10^4	5.51×10^{12}	0.9967

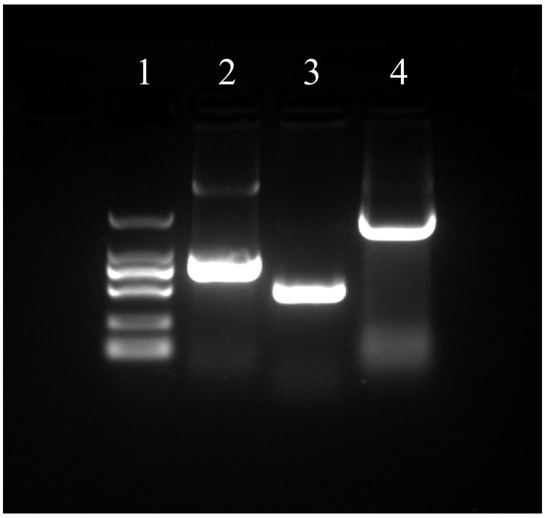

Supplementary Figure S1 PCR product detection of target genes in the strain DN1 with different genotypes. Genomic DNA from the DN1 was the template. Hole 1 was the DNA Marker, hole 2 was gene encoding alpha subunit deletion mutant (△*rhdA*), hole 3 was gene encoding beta subunit deletion mutant (△*rhdB*), and hole 4 was wide-type gene of RHD. Primers used to construct mutants shown in Table 1.

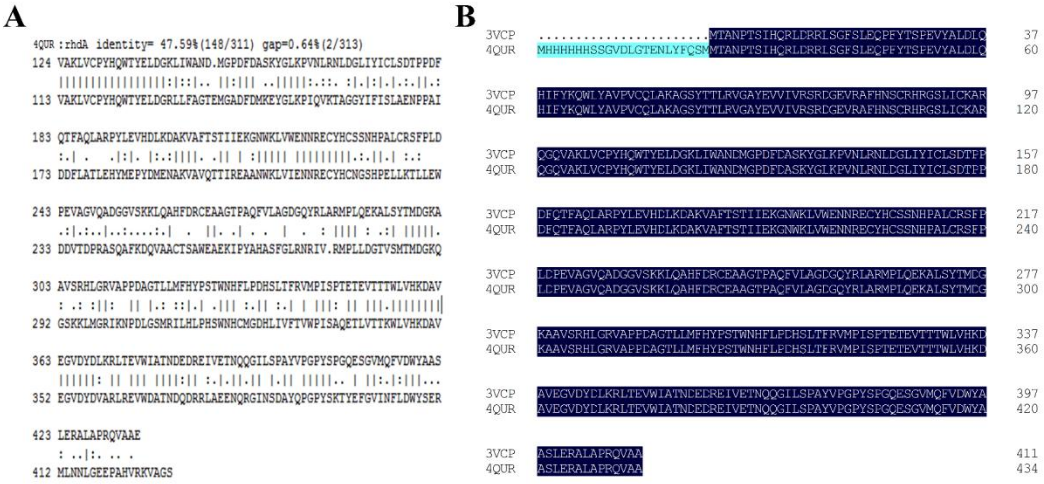

Supplementary Figure S2 Sequence alignment of amino acid residues of the template 4QUR and the targeted alpha subunit of RHD (A), and sequence alignment of amino acid residues of the template 3VCA and 4QUR (B).

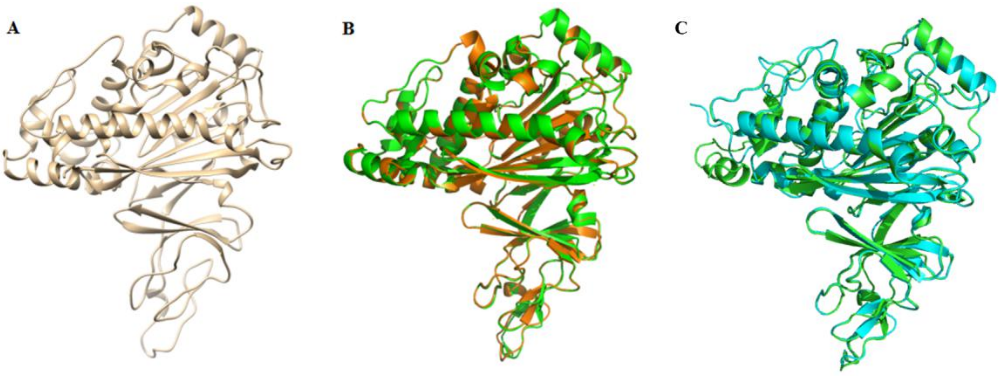

Supplementary Figure S3 The modeled crystal structure of alpha subunit of RHD using a Ramachandran plot (A), schematic of superposition between the modeling alpha subunit of RHD (orange ribbon) and template 4QUR (green ribbon) (B), and schematic of superposition between template 4QUR (green ribbon) and template 3VCA (blue ribbon) (C).

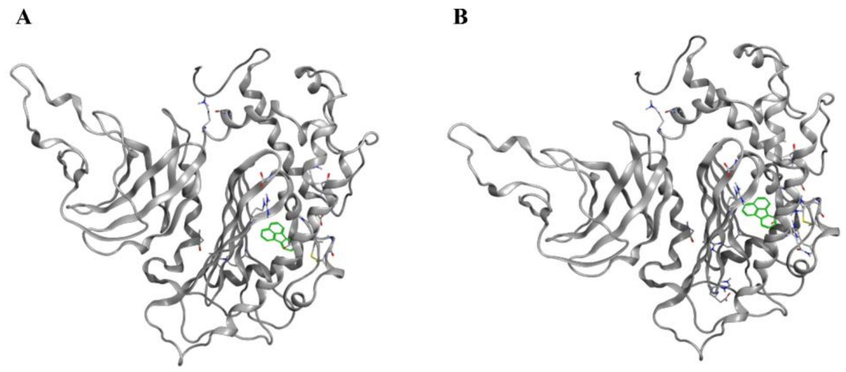

Supplementary Figure S4 The combination state of fluoranthene in the active site of RHD for the homology modeling according to the crystal structure of the template 4QUR (A) and 3VCA (B).

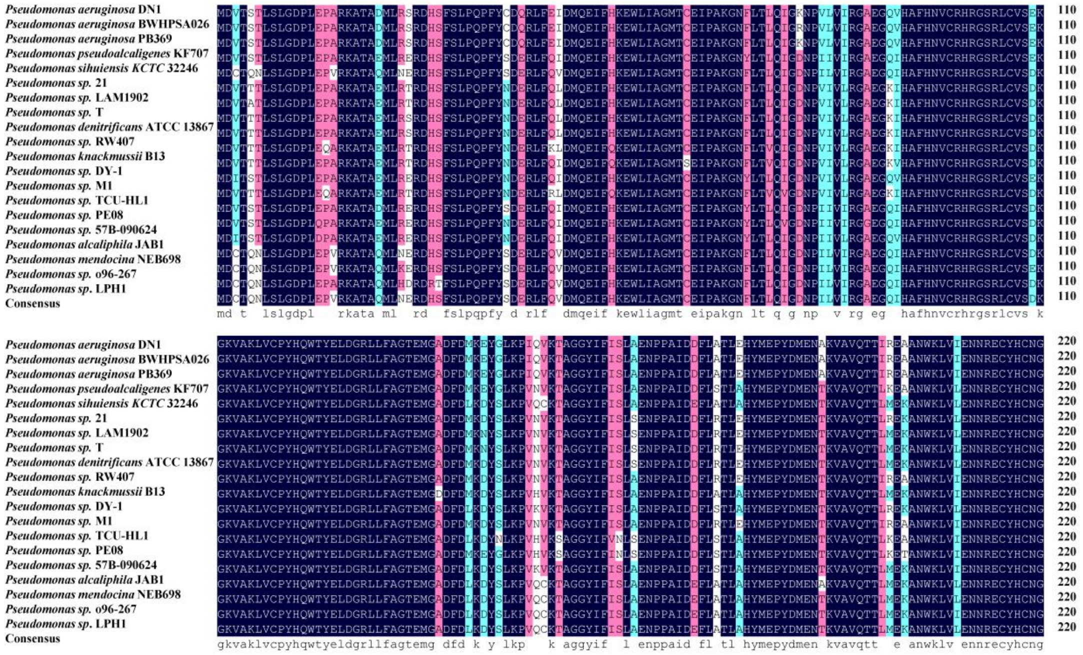

Supplementary Figure S5 The multiple sequence alignment of amino acid residues existed in alpha subunit of RHD from different strains of the genus *Pseudomonas* (*Shading* with *different colors* in all the sequences represent the similarity of side chains of amino acid residues in the alignment. The conserved amino acid residues in all of the sequences are indicated with a *blue-black* background, and the other colors is in direct proportion to homologous comparison. *Pink colors* represent 75 % homology of amino acid, *wathet blue colors* represent 50 % homology of amino acid, and *white colors* represent 30 % homology of amino acid).

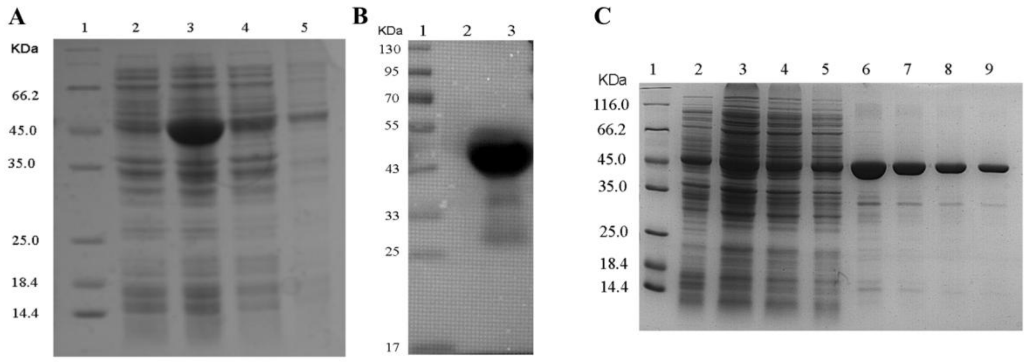

Supplementary Figure S6 Expression and purification of recombinant RhdA protein. A: Soluble analysis of RhdA, B: The prokaryotic expression products were detected by Western Blot, C: Mass expression and Ni column purification of RhdA.

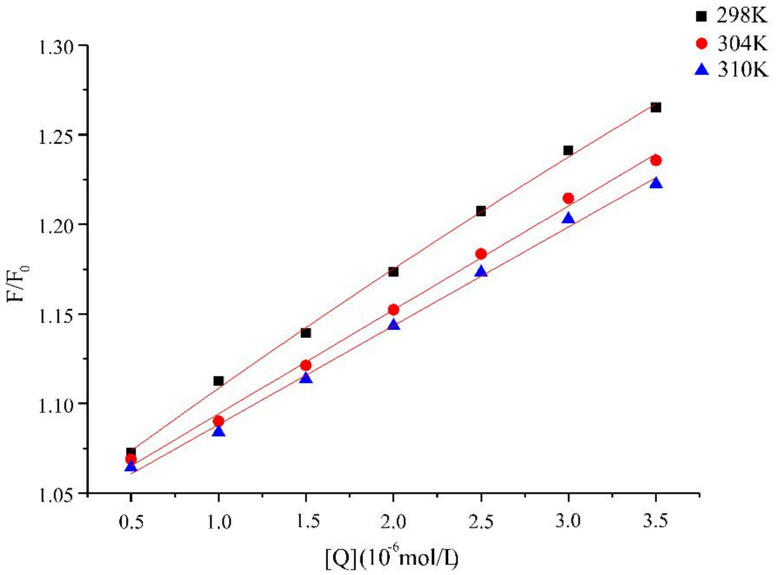

Supplementary Figure S7 The Stern-Volmer plots of RHD alpha subunit quenched by fluoranthene at different temperatures (298, 304 and 310 K).

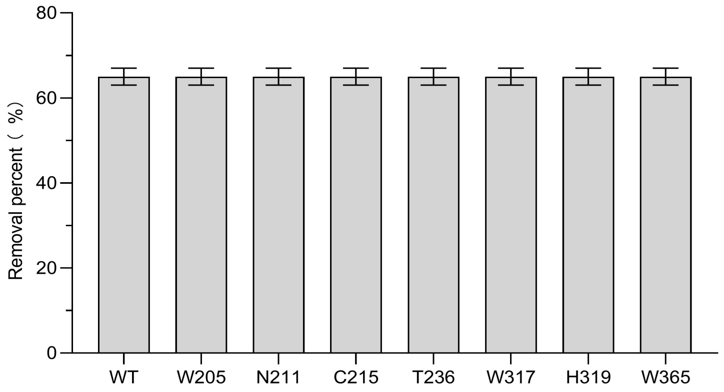

Supplementary Figure S8 Degradation efficiency of the wide-type DN1 (WT) and its mutants of other hydrophilic amino acid residues around the active site of RHD alpha subunit.
